# The Ambulance Dog in War

**Published:** 1914-10-24

**Authors:** 


### The Ambulance Dog in War.

MAJOR RICHARDSON'S EXPERIMENTS.

There is abundant evidence that the dog from the earliest times has played its part in war, for we find Strabo mentioning the use of the bloodhound in the wars of Gaul, while historians tell us that the Earl of Essex in his campaign in Ireland in the reign of Queen Elizabeth used some 800 of these animals. It is, however, probable that the dogs in the incidents recalled were used solely in connection with fighting, and it has been left to modern times to train them for ambulance purposes. So far as this country is concerned it is to Major Richardson that the existence of the ambulance dog is chiefly due, and through his kindness we are enabled to give a few notes of their use in modern warfare.

Dogs for ambulance work are now largely used in Holland, being attached to the Grenadier regiments at The Hague, and a training school has recently been established there under the presidency of the Prince Consort of the Netherlands. Sweden endeavours to stimulate interest in the use of dogs by holding trials at certain intervals at Stockholm; the number of dogs used by the military authorities in Russia is rapidly increasing, while an excellent and elaborate system is in vogue in the Belgian Army.

Major Richardson, who, during the recent Morocco, Tripoli, and Balkan campaigns, personally studied the uses of dogs for military purposes, and who has bred and supplied numerous animals to the Admiralty, Norfolk, Durham Light Infantry, Gordon Highlanders, and Gurkha regiments, points out that the chief work of the ambulance dog is to seek for the wounded, and lays stress upon the difficulty often experienced in recovering the fallen, especially in a country where the battlefield may be an expanse of jungle or undergrowth, or when circumstances necessitate that the search for wounded must be largely undertaken at night. In this connection he quotes some interesting words of Count Persidsky, who says: "In finding the missing and wounded, with which the millet fields are strewn, nothing succeeded like our pack of seven dogs. The English ones are especially intelligent. In our last engagement twenty-three men were found in unsuspected places."

A suggestion worthy of serious consideration, made some two years ago, that each Red Cross and St. John Ambulance Detachment should procure and train an ambulance dog has, we fear, not been carried out in its entirety, though a number of "Red Cross" dogs are being utilised to good advantage in the present war. Major Richardson, in a paper read before the Royal United Service Institution in 1912 on the selection of suitable dogs, pointed out that intelligence, hearing, and scent were, of course, of primary importance; that very heavily coated dogs are not advisable on account of the heat in hot climates, and the difficulty of keeping such animals free from insect life; the Airedale was the best in this country, as it possessed the qualities required as to intelligence, etc., as well as being of medium size, hardy, and faithful. Only about one in twenty animals ultimately prove to have been worth training.

